# Optimal Hydrogen Production Coupled with Pollutant Removal from Biodiesel Wastewater Using a Thermally Treated TiO_2_ Photocatalyst (P25): Influence of the Operating Conditions

**DOI:** 10.3390/nano8020096

**Published:** 2018-02-09

**Authors:** Pimsuda Pansa-Ngat, Trin Jedsukontorn, Mali Hunsom

**Affiliations:** 1 Fuels Research Center, Department of Chemical Technology, Faculty of Science, Chulalongkorn University, Bangkok 10330, Thailand; pimsuda.pansangat@yahoo.com (P.P.-N.); Trinatabo_jed@hotmail.com (T.J.); 2 Center of Excellence on Petrochemical and Materials Technology (PETRO-MAT), Chulalongkorn University, Bangkok 10330, Thailand; 3 Associate Fellow of Royal Society of Thailand (AFRST), Bangkok 10300, Thailand

**Keywords:** biodiesel wastewater, H_2_ production, titanium dioxide, light conversion efficiency

## Abstract

This work aimed to produce hydrogen (H_2_) simultaneously with pollutant removal from biodiesel wastewater by photocatalytic oxidation using a thermally-treated commercial titanium dioxide (TiO_2_) photocatalyst at room temperature (~30 °C) and ambient pressure. The effects of the operating conditions, including the catalyst loading level (1–6 g/L), UV light intensity (3.52–6.64 mW/cm^2^), initial pH of the wastewater (2.3–8.0) and reaction time (1–4 h), on the quantity of H_2_ production together with the reduction in the chemical oxygen demand (COD), biological oxygen demand (BOD) and oil and grease levels were explored. It was found that all the investigated parameters affected the level of H_2_ production and pollutant removal. The optimum operating condition for simultaneous H_2_ production and pollutant removal was found at an initial wastewater pH of 6.0, a catalyst dosage of 4.0 g/L, a UV light intensity of 4.79 mW/cm^2^ and a reaction time of 2 h. These conditions led to the production of 228 μmol H_2_ with a light conversion efficiency of 6.78% and reduced the COD, BOD and oil and grease levels by 13.2%, 89.6% and 67.7%, respectively. The rate of pollutant removal followed a pseudo-first order chemical reaction with a rate constant of 0.008, 0.085 and 0.044 min^−1^ for the COD, BOD and oil and grease removal, respectively.

## 1. Introduction

Biodiesel is recognized as an alternative fuel that can be used in compression-ignition diesel engines, either in a pure form or blended with petroleum diesel, with little or no modifications due to its high cetane number and lubricity [1]. In addition, it is safer and cleaner than fossil fuel-derived diesel because it has a high flash point and emits a lower level of sulfur dioxide, hydrocarbons, particulates, polycyclic aromatic hydrocarbons and carbon monoxide [2].

Currently, most biodiesel is derived from the chemical modification, specifically the transesterification (or alcoholysis), of vegetable oil or animal fat in the presence of methanol with acid and basic catalysts [3]. This process allows the use of higher (e.g., C_4_) alcohols in the process and produces a less polar and corrosive fatty acid methyl ester mixture with reduced cloud and pour points [4]. However, for every 100 L of biodiesel produced, approximately 20 L of wastewater are generated, which contains a high content of several impurities, such as saturated and unsaturated free fatty acids (FFA), glycerol, methanol, water and soap [5,6]. In Thailand, it is expected that the biodiesel consumption will grow by 8% to 1.27 × 10^9^ L in 2017, compared to 1.18 × 10^9^ L in 2014. This is because the prevailing low prices for diesel encourage diesel vehicle users (mainly trucks or trailers) to switch to diesel from compressed natural gas and also stimulates the increased use of diesel among smaller vehicles (e.g., pick-up trucks) [7]. This means that at least 2.54 × 10^8^ L of biodiesel wastewater will be produced, which will have to be managed and treated prior to discharge into the environment. Currently, several processes have been developed to treat or improve the properties of biodiesel wastewater, such as biological [5], physical [8,9], chemical [10,11,12], electrochemical [6,13,14,15,16] and combined chemical-electrochemical [17,18] processes. However, most of these processes can function only in organic pollutant degradation/removal and leave the chemical substances in the treated wastewater or generate large volumes of low density sludge, which still leaves economic and environmental problems with its disposal.

A new wastewater treatment process, known as the photocatalytic oxidation process, is able to remove the organic pollutants from biodiesel wastewater simultaneously with the production of hydrogen (H_2_), a green fuel. In this process, when the photocatalyst is irradiated with light having a photon energy equal to or greater than its band gap energy, the electron (*e*^−^) is excited from the valence band to the conduction band, leaving a hole (*h*^+^) in the valence band [19]. The photogenerated *h*^+^ is able to oxidize the surface-bonded water molecules to produce highly reactive hydroxyl radicals (OH^•^), which are able to oxidize the organic pollutants in the biodiesel wastewater, while the generated *e*^−^ can further react with a proton (H^+^) to form gaseous H_2_ [20,21,22]. In addition, excess photogenerated *h*^+^ can react irreversibly with organic molecules in the wastewater, resulting in a suppressed *e*^−^–*h*^+^ recombination and/or a reduced reverse reaction between O_2_ and H_2_ [23]. The mechanism for organic substances denoted as RCH_2_OH and R′CH_2_OH degradation to H_2_ via the photocatalytic oxidation has been proposed, as shown in Equations (1)–(6) [20].

RCH_2_OH → H^+^ + RCH_2_O^−^ (1)

RCH_2_O^−^ + *h*^+^ → RCH_2_O^•^ (2)

(3) $$R{\mathrm{CH}}_{2}O^{•}+R^{\prime }{\mathrm{CH}}_{2}\mathrm{OH}\;\to \;R{\mathrm{CH}}_{2}\mathrm{OH}+R^{\prime }\overset{•}{C}\mathrm{HOH}$$

(4) $$R^{\prime }\overset{•}{C}\mathrm{HOH}+h^{+}\;\to \;H^{+}+R^{\prime }\overset{•}{C}{\mathrm{HO}}^{•}\;\to \;R^{\prime }\mathrm{CHO}$$

R′CHO + HO^•^ → [R′COOH]^−^ + H^+^ (5)

2H^+^ + 2*e*^−^ → H_2_ (6)

where RCH_2_OH and R′CH_2_OH are the organic substances contained in wastewater.

If the complete organic pollutant degradation is achieved, the carbon dioxide (CO_2_) is obtained as the co-product with H_2_, as shown in Equation (7) [20].

[R′COOH]^−^ + *h*^+^ → R′H + CO_2_ (7)

According to our published work, it was found that the simultaneous H_2_ production and pollutant removal from biodiesel wastewater could be achieved by UV photocatalytic oxidation with titanium dioxide (TiO_2_) using a 3.3-fold dilution of the wastewater [24], and the crystal structure of TiO_2_ markedly affected the rate of H_2_ production and pollutant removal. The mixed anatase-rutile phase crystal structure of TiO_2_ photocatalysts exhibited a higher photocatalytic activity than that with a single rutile crystal structure, due to the co-presence of rutile and anatase phases of TiO_2_. Thus, in order to achieve a high efficiency of H_2_ production together with pollutant removal, the optimum operating condition was determined at ambient temperature and pressure using a mixed anatase-rutile phase crystal structure TiO_2_. The novelty of this work is the determination of optimum operating condition to produce the H_2_ simultaneously with pollutant removal from real biodiesel wastewater, which had never been studied before. The information on the optimum condition for simultaneous H_2_ production and wastewater treatment will have a beneficial effect on the economics of the biodiesel production plants.

## 2. Experimental Section

### 2.1. Preparation of the Photocatalyst and Characterization

The photocatalyst used in this work was prepared by a thermal treatment of a commercial TiO_2_ (P25, Degussa, St. Louis, Mo, USA) in air at 400 °C for 3 h in order to achieve the formation of micropore structures and eliminate some impurities [24]. Its morphology and textural property were respectively characterized by the X-ray diffractometer (XRD, D8 Discover-Bruker AXS, Billerica, MA, USA) and a surface area analyzer (Quantachrome Instruments, Autosorb-1, Boynton Beach, FL, USA) according to the Brunauer–Emmett–Teller (BET) method. The optical absorption spectra was analyzed by a UV-visible spectrophotometer (UV-2550, Shimadzu, Kyoto, Japan) at a wavelength range of 350–550 nm. The point of zero charge (PZC) of the utilized photocatalyst was obtained by dispersing 0.4 g of photocatalyst with 20 mL of 0.1 M KNO_3_ (Ajax Finechem, Taren Point, Australia) at room temperature (~30 °C). The initial pH of KNO_3_ was adjusted to a value between 2 and 12 by adding 0.1 M HNO_3_ (QReC, Chonburi, Thailand) or 0.1 M KOH (QReC). The suspended solution was agitated at a constant rate of 120 rpm for 24 h. The PZC value was determined by plotting the initial and final pH of the KNO_3_ solution [25].

### 2.2. Simultaneous H_2_ Production and Pollutant Removal

**Figure 1:**
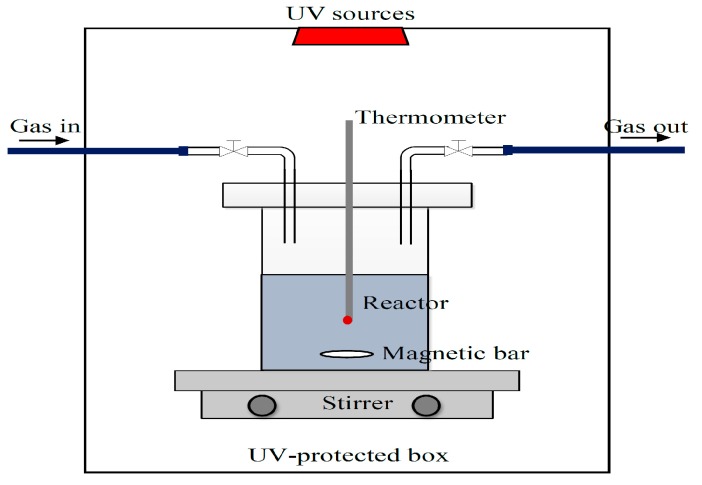
Scheme of the photoreactor.

The biodiesel wastewater used in this work was collected from the biodiesel industry in Thailand. Prior to use, some contaminants were preliminary removed by acidification with sulfuric acid (H_2_SO_4_; 98%, Fisher, Guangzhou, China) to a pH of around 1–2 [14], whereupon the wastewater automatically separated into the two phases of an oil-rich top layer and water-rich bottom layer or the pretreated wastewater that was then separated by slow decantation. The pretreated wastewater was then diluted 3.3-fold with distilled water [24] and subjected to the photoreactor to produce H_2_ simultaneously with pollutant removal (Figure 1). In each experiment, approximately 150 mL of pretreated wastewater was filled in a hollow closed Pyrex glass cylinder and put in the middle of a UV-protected box (0.68 m × 0.68 m × 0.78 m). The required dosage of the photocatalyst (range of 1.0–6.0 g/L) was added under a constant agitation rate of 250 rpm. To eliminate the air from the system, argon (Ar) gas was flushed at constant flow rate of 500 mL/min for an hour. The reactor was then illuminated by a 120-W UV high-pressure mercury lamp (RUV 533 BC, Holland, The Netherlands) set on the roof of the UV-protected box [24] at the selected light intensity (range of 3.52–6.64 mW/cm^2^) for the desired reaction time (14 h). As the experiment progressed, the photogenerated gas was quantitatively characterized by gas chromatography (GC 2014, Shimadzu, Kyoto, Japan) coupled with a thermal conductivity detector and molecular sieve 5A column. The liquid product was also collected and centrifuged on a KUBOTA KC-25 digital laboratory centrifuge (Tokyo, Japan) to separate the solid catalyst, and the supernatant was analyzed for the pollutant contents in terms of the levels of the biological oxygen demand (BOD), chemical oxygen demand (COD), oil and grease, total dissolved solids (TDS) and total suspended solids (TSS) according to the standard method [26]. In addition, the soap content was analyzed according to the modified version of the American Oil Chemists’ Society (AOCS) Method Cc 17–79 [27]. The free fatty acid (FFA) content was determined from the ratio of acid value to 2.19 using the potentiometric titration according to the ASTM D 664 [27,28].

## 3. Results and Discussion

### 3.1. Properties of Photocatalyst

The morphology and properties of the utilized photocatalyst were already mentioned in our previous work [24]. It had a crystallite size, anatase content, band gap energy and point of zero charge (PZC) of 26.4 nm, 87.4%, 3.18 eV and 6.8, respectively. In addition, it had a BET surface area, micropore volume and mesopore volume of 51.8 m^2^/g, 0.022 cm^3^/g and 0.240 cm^3^/g, respectively.

### 3.2. Properties of Fresh and Acid-Pretreated Biodiesel Wastewater

**Figure 2:**
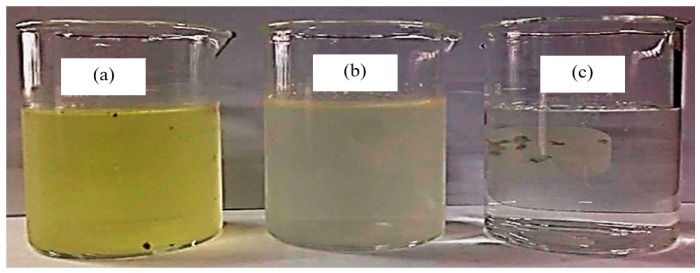
Appearance of (**a**) fresh wastewater; (**b**) acid-pretreated wastewater and (**c**) treated wastewater by photocatalytic oxidation.

The fresh biodiesel wastewater was a pale yellow solution (Figure 2a). Quantitatively, it contained low FFA content and a high content of soap and glycerol (Table 1). In terms of the wastewater quality, it was slightly acidic (pH 4.07–4.12) and contained very high COD and TDS contents compared with the BOD, oil and grease and TSS contents. That is, the COD, BOD, oil and grease, TDS and TSS levels were some 296–367-, 10–20-, 44–125-, 2.5–4.5- and 3.5–4.2-fold greater, respectively, than the respective acceptable values set by the Thai Government for discharging into the environment. The color and property of the fresh wastewater was slightly improved after the H_2_SO_4_ pretreatment step (Figure 2b). Quantitatively, the soap content was markedly reduced, while the FFA and glycerol contents increased over seven- and 1.2-fold, respectively, (Table 1). This is due to the (i) fast protonation of the fatty acid salts by H^+^ dissociated from the utilized H_2_SO_4_, resulting in the formation of the less polar and water-insoluble free fatty acids (FFAs) [19]; and (ii) the combination of some dissociated H^+^ with the biodiesel leading to the formation of water-insoluble free fatty acid methyl ester (FAMEs) [18]. Nevertheless, the H_2_SO_4_-pretreated wastewater still contained COD, BOD, oil and grease, TDS and TSS contents that were greater than the acceptable value of around 152–241-, 3.5–7.7-, 22.4–89.2-, 3.8–4.2- and 0.8–2.0-fold, respectively.

### 3.3. Simultaneous H_2_ Production and Pollutant Removal

#### 3.3.1. Requirements for Both UV Irradiation and the Photocatalyst

**Figure 3:**
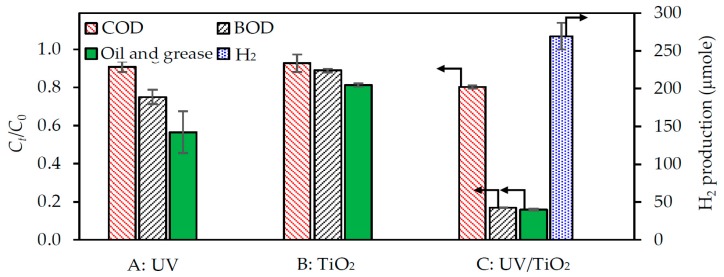
Comparison of pollutant removal and H_2_ production of the 3.3-fold diluted acid-pretreated biodiesel wastewater after a 4-h treatment with (**A**) UV light at an intensity of 4.79 mW/cm^2^; (**B**) photocatalyst at 4.0 g/L and (**C**) UV/TiO_2_ at a dosage of 4.0 g/L and UV light intensity of 4.79 mW/cm^2^.

The simultaneous H_2_ production and pollutant removal was evaluated with (i) UV irradiation only (4.79 mW/cm^2^, no photocatalyst); (ii) the photocatalyst only (4.0 g/L, no UV light) and (iii) in the presence of both UV light and the photocatalyst (4.79 mW/cm^2^ and 4.0 g/L, respectively) with the 3.3-fold diluted acid-pretreated wastewater at an initial pH of around 2.0 at 4 h. As exhibited in Figure 3, the levels of BOD, COD and oil and grease were slightly reduced (7% for COD, 12% for BOD and 18% for oil and grease) in the presence of the photocatalyst only or in the presence of the UV light only (9% for COD, 25% for BOD and 44% for oil and grease), without any detected H_2_ production. However, in the presence of both the UV irradiation and photocatalyst, these pollutants were more markedly reduced (20% for COD, 83% for BOD and 84% for oil and grease) along with H_2_ production (~270 µmol). This is probably attributed to the different mechanisms for H_2_ production and pollutant removal. That is, the removal of pollutant molecules in the presence of the UV light only occurred by the breakdown of pollutant molecules via the hydroxyl radicles (OH^•^) generated from the water photolysis (Equation (8)) [29]. However, this reaction is a poor source of radicals, and in the oxidation process, some intermediates absorbing part of the radiation are generated, causing a decrease in the photooxidation kinetics of the pollutants. For the presence of photocatalyst without UV irradiation, the removal of pollutant molecules was probably caused by the adsorption process between the positive surface charge of the photocatalyst (${\mathrm{TiOH}}_{2}^{+}$) and the negative charge of pollutant molecules. These are also the reasons why no H_2_ was produced in these two conditions.

H_2_O + *hv* → H^•^ + OH^•^ (8)

In the presence of both UV irradiation and photocatalyst, the possible mechanism for H_2_ production and pollutant removal via this condition might follow the reactions shown in Equations (1)–(6). The complete organic pollutant degradation via the photocatalytic oxidation released the H_2_ as the main product with the carbon dioxide (CO_2_) as the co-product, as shown in Equation (7). However, in our experiment, no trace of CO_2_ was detected, suggesting that a complete photooxidation of the organic substances in biodiesel wastewater was not achieved, leaving intermediate species that would be accounted for in terms of the residual COD, BOD and oil and grease levels in the treated wastewater.

#### 3.3.2. Effect of the Photocatalyst Loading

**Figure 4:**
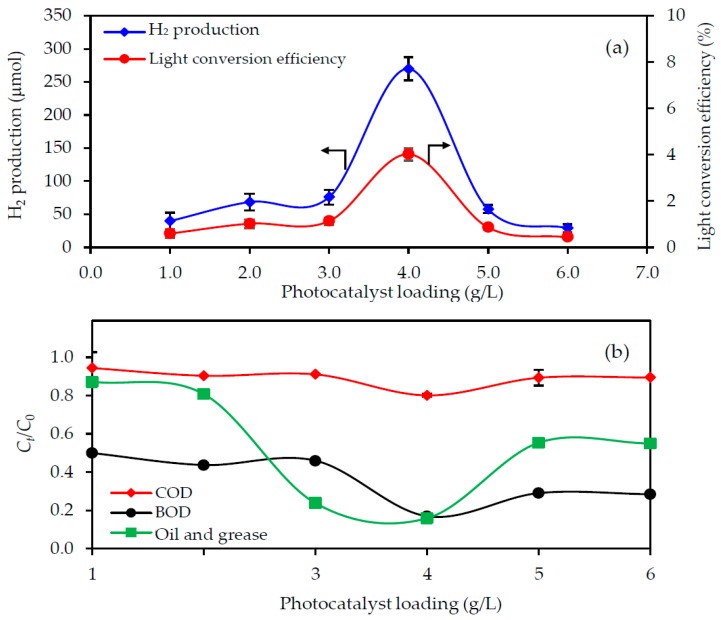
Effect of the photocatalyst loading on the level of (**a**) H_2_ production with light conversion efficiency and (**b**) pollutant removal from the 3.3-fold diluted acid-pretreated biodiesel wastewater at an initial pH of 2.3 with a UV light at intensity of 4.79 mW/cm^2^ for 4 h.

The effect of varying the photocatalyst loading (1.0–6.0 g/L) on the H_2_ production and pollutant removal was evaluated using the 3.3-fold diluted acid-pretreated biodiesel wastewater at an initial pH of 2.3, UV light intensity of 4.79 mW/cm^2^ and reaction time of 4 h. The maximum H_2_ production level (Figure 4a) and lowest residual level of COD, BOD, oil and grease contents (Figure 4b) were all obtained with a photocatalyst loading of 4.0 g/L. The increased H_2_ production and COD, BOD and oil and grease removal levels with an increasing photocatalyst loading from 1.0–4.0 g/L presumably reflects the increased number of available adsorption sites, supplying more molecules in the biodiesel wastewater to perform the reaction. However, the decreased H_2_ production and pollutant removal levels with photocatalyst loading above 4.0 g/L might be attributed to the formation of the self-shading effect [30,31], in which catalyst particles reduce the light intensity in the wastewater of the photocatalyst particles, owing to their low photo-excitation centers. Another possible reason is the light absorption and scattering according to the Beer–Lambert law, resulting in a lower effective quantity of incident light on the catalyst surface. Regardless, the optimal level of photocatalyst in this study was 4.0 g/L.

In terms of the light conversion efficiency, the efficiencies calculated from the ratio of the total energy value of the obtained H_2_ to the total energy input to the photoreactor by light irradiation, derived from Equation (9) [32], were 0.59%, 1.02%, 1.13%, 4.01%, 0.86% and 0.43% for a photocatalyst loading of 1.0, 2.0, 3.0, 4.0, 5.0 and 6.0 g/L, respectively (Figure 4a). This emphasized that the photocatalyst loading of 4.0 g/L was the optimum level, since it exhibited the highest light conversion efficiency:

(9) $$\eta =\frac{33.61\rho_{H_{2}}V_{H_{2}}}{IAt}\times 100$$

where $V_{H_{2}}$ is the volume of produced H_2_ (L), $\rho_{H_{2}}$ is the density of the produced H_2_ (g/L), *I* is the light intensity (W/m^2^), *A* is the irradiated area (m^2^) and *t* is the duration of H_2_ production (h).

#### 3.3.3. Effect of the UV Light Intensity

**Figure 5:**
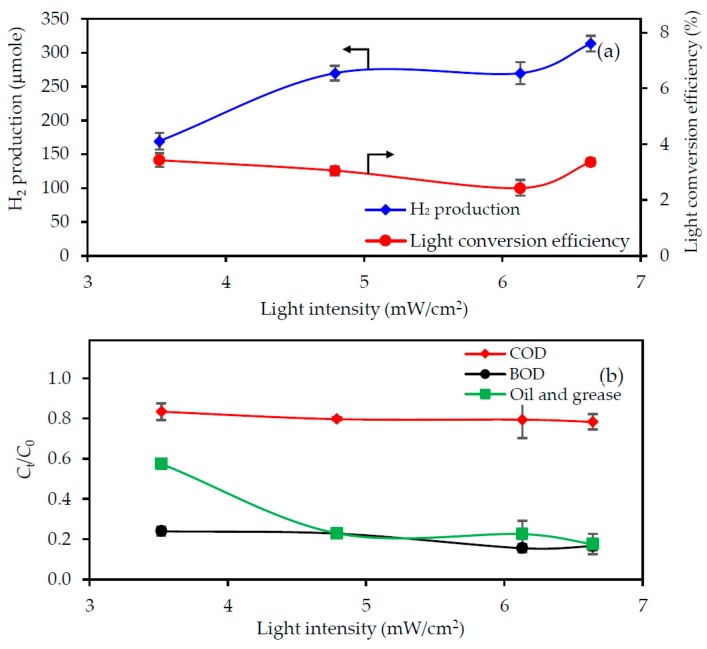
Effect of the UV light intensity on the level of (**a**) H_2_ production with light conversion efficiency and (**b**) pollutant removal from the 3.3-fold diluted acid-pretreated biodiesel wastewater at an initial pH of 2.3 with a photocatalyst loading of 4.0 g/L for 4 h.

Figure 5 shows the effect of varying the UV light intensity (3.52–6.64 mW/cm^2^) on the H_2_ production and pollutant removal levels from the 3.3-fold diluted acid-pretreated biodiesel wastewater at an initial pH of 2.3 with photocatalyst loading of 4.0 g/L for 4 h. Increasing the light intensity from 3.52–4.79 mW/m^2^ increased the H_2_ production from 169–270 μmol (Figure 5a), decreased the levels of COD and BOD only slightly and oil and grease more markedly in the wastewater (Figure 5b). Further increasing the UV light intensity above 4.79 mW/cm^2^ did not markedly change the amount of produced H_2_, suggesting the saturation of H_2_ production in the presence of high light intensity. The saturation of H_2_ production has been observed previously for the photobiological production of H_2_ at a high light intensity using malate and sodium glutamate as the carbon and nitrogen sources, respectively [32,33]. The low pollutant removal and H_2_ production levels at a light intensity of less than 4.79 mW/cm^2^ likely reflected an insufficient light penetration onto the photocatalyst surface. There was no increase in pollutant removal or H_2_ production at UV light intensities greater than 4.79 mW/m^2^, which is probably attributed to (i) a high *e*^−^–*h^+^* recombination rate compared with the surface reaction when there was an extremely high *e^−^*–*h^+^* generation rate [20] and/or (ii) the limitation of available photocatalyst surface to absorb a large quantity of incident light to perform the reaction.

The light conversion efficiencies were 3.43%, 3.05%, 2.64% and 3.13% in the presence of a UV light intensity of 3.52, 4.79, 6.13 and 6.64 mW/cm^2^, respectively. The slight change in the light conversion efficiency as the light intensity increased indicated the limitation of the photocatalyst surface to absorb the incident light. In other words, the efficiency of H_2_ production and pollutant removal at a high light intensity range was controlled by the surface reaction instead of the light intensity.

#### 3.3.4. Effect of the Initial Wastewater pH

**Figure 6:**
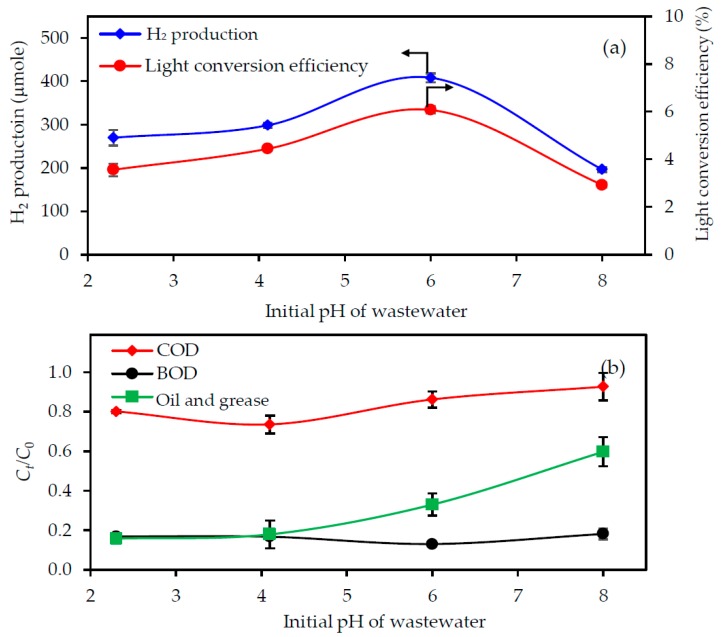
Effect of the initial pH of the wastewater on the level of (**a**) H_2_ production with light conversion efficiency and (**b**) pollutant removal from the 3.3-fold diluted acid-pretreated biodiesel wastewater with a UV light intensity of 4.79 mW/cm^2^ and photocatalyst loading of 4.0 g/L for 4 h.

The effect of the initial pH of the wastewater on the H_2_ production rate and pollutant removal was explored using the 3.3-fold diluted acid-treated biodiesel wastewater with a UV light intensity of 4.79 mW/cm^2^, photocatalyst loading of 4.0 g/L and a reaction time of 4 h. As exhibited in Figure 6a, increasing the initial pH of the wastewater from 2.3–6.0 enhanced the H_2_ production level from 270–408 μmol. Further raising the initial pH of the wastewater to 8.0 decreased the H_2_ production almost two-fold. For the pollutant removal, increasing the initial pH from 2.3–8.0 insignificantly affected the BOD removal, but above pH 4.0, it decreased the oil and grease removal (Figure 6b). The highest COD removal was observed at an initial wastewater pH of 4.1 and decreased with increasing initial pH. The light conversion efficiency of the wastewater with an initial pH of 2.3, 4.1, 6.0 and 8.0 was 3.56%, 4.44%, 6.07% and 2.92%, respectively. Thus, the appropriate initial pH of the wastewater for H_2_ production and pollutant removal were not the same, pH 6.0 being optimal for H_2_ production, but pH 4.1 for pollutant removal. This might be attributed to the effect of the PZC and agglomeration behavior of the utilized photocatalyst with the change in the wastewater pH. The surface of the utilized photocatalyst is positively charged (TiOH_2_^+^) in wastewater with a pH less than the PZC of photocatalyst, while it is negatively charged (TiO^−^) in wastewater with a pH higher than the PZC [34]. Under an acid condition, the electrostatic repulsion between the positively-charged surface of photocatalyst and H^+^ would decrease as the initial pH of the wastewater increased, resulting in an agglomeration of the catalyst particles. In other words, the catalyst was well-dispersed (low agglomeration) at a pH less than PZC and poorly-dispersed (high agglomeration) at a pH close to the PZC. This agglomeration brought the catalyst nanoparticles in close contact with each other through the grain boundaries, allowing the migration of *e^−^* and *h*^+^ to an adjacent particle hopping through the grain boundary [35], resulting in a low probability of *e^−^*–*h*^+^ recombination, as well as the high production of H_2_ according to Equation (7). Thus, the wastewater with an initial pH of 6.0 provided a higher H_2_ production level than that with an initial pH of 4.1. However, the agglomeration of the T_400_ particles reduced the available surface area to adsorb the pollutant molecules to degrade with the photogenerated *h*^+^ and OH^•^, as shown in Equations (5) and (6), resulting in a lower level of pollutant removal in the presence of wastewater with an initial pH of 6.0 compared to that with a pH of 4.1. The H_2_ production and pollutant removal rates decreased markedly, at an initial pH of 8.0, which was probably due to the electrostatic repulsion between the negative charges on the photocatalyst surface and the lone-pair electron of the pollutant molecules, which would inhibit the adsorption of pollutant molecules and so result in a decreased photocatalytic activity. Although the wastewater with an initial pH of 6.0 had a 1.92-fold lower COD removal level than that with initial pH of 4.1, it provided a 1.4-fold higher H_2_ production level. The final pH of the treated wastewater was 4.74 and 8.76 for the wastewater with an initial pH of 4.1 and 6.0, respectively. Thus, an initial pH of wastewater of 6.0 was selected as the optimum pH for the simultaneous H_2_ production and pollutant degradation.

#### 3.3.5. Effect of the Operating Time and Reaction Rate

**Figure 7:**
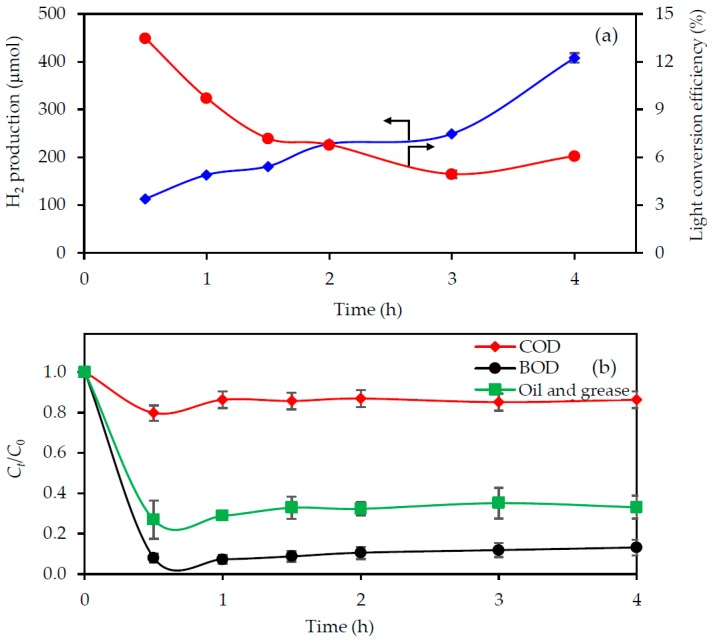
Effect of the reaction time on the level of (**a**) H_2_ production with light conversion efficiency and (**b**) pollutant removal from the 3.3-fold diluted acid-pretreated biodiesel wastewater with an initial pH of 6.0 using a photocatalyst loading of 4.0 g/L and UV light intensity of 4.79 mW/cm^2^.

Figure 7 exhibits the variation in the H_2_ production and decrease in the COD, BOD and oil and grease levels via the photocatalyst-UV light-mediated photocatalytic oxidation of the 3.3-fold diluted acid-pretreated biodiesel wastewater at an initial pH of 6.0, a photocatalyst loading of 4.0 g/L and UV light intensity of 4.79 mW/cm^2^. The amount of H_2_ produced increased linearly with the reaction time, while the light conversion efficiency decreased with increasing reaction times up to 3.0 h and then slightly increased at 4 h (Figure 7a). With respect to the COD, BOD and oil and grease levels, their concentration decreased rapidly during the first 30 min of reaction time and then remained broadly constant afterwards (Figure 7b).

The rate of COD, BOD and oil and grease removal was dependent on the amount of the reactive oxidizing species (ROS), including the *h*^+^ and OH^•^, generated in the system and the concentration of pollutants, as demonstrated in Equations (5) and (6). Thus, it can be written as Equation (10);

(10) $$\frac{dC}{dt}=-k^{\prime }C[\mathrm{ROS}]$$

where *k′* is the rate constant, *C* is the concentration of COD, BOD and oil and grease and [ROS] is the concentration of the generated ROS.

Since the quantity of ROS was generated constantly at a given set of experimental conditions, their concentration did not limit the rate of pollutant degradation. Therefore, Equation (10) can be written as Equation (11);

(11) $$\frac{dC}{dt}=-kC$$

where *k* is the pseudo-first order rate constant of pollutant degradation.

Integrating Equation (11) with the boundary condition at *t* = 0, *C* = *C*_0_ and *t* = *t*, *C* = *C_t_* and rearrangement yields Equation (12);

(12) $$C_{t}=C_{0}\exp(-kt)$$

The pseudo-first order rate constant (*k*) can be determined from this relationship by plotting ln(*C_t_*/*C*_0_) against *t*. Accordingly, the pseudo-first order rate constants of COD, BOD and oil and grease removal were found to be 0.008, 0.085 and 0.044 min^−1^, respectively. The rate constant of COD removal was lower than those of BOD and oil and grease removals by around 10.6- and 5.5-fold, respectively. This is probably due to the very high initial COD concentration compared with that of BOD and oil and grease. Another possible reason is the degradation of long organic molecules to short-chain non-biodegradable molecules, which would then be accounted for in terms of the residual COD level in the treated wastewater.

After the photocatalytic oxidation at optimum condition (3.3-fold dilution, initial pH of 6.0, photocatalyst loading of 4.0 g/L, UV light intensity of 4.79 mW/cm^2^), the clear wastewater was obtained as shown in Figure 2c. There, soap and FFAs were decreased to 0.09–1.07 wt % and 0.02–0.04 wt %, respectively, without any detectable trace of glycerol (Table 1). The pH and BOD levels were reduced to within the acceptable Thai standard, whilst the levels of COD, oil and grease, TDS and TSS were still greater than the acceptable values by around 61.8–62.3-, 13.7–16.2-, 2.6–3.0- and 0.9–1.3-fold, respectively. Thus, a more extensive study is still required and currently underway to improve the photocatalyst’s morphology in order to increase the rate of H_2_ production together with pollutant removal, and the obtained results will be reported soon.

## 4. Conclusions

The optimum conditions for the simultaneous H_2_ production and pollutant removal from acidified biodiesel wastewater by photocatalytic oxidation with photocatalyst, a thermally-treated commercial TiO_2_ photocatalyst, were explored at room temperature (~30 °C) and ambient pressure. The photocatalyst loading level, UV light intensity, initial pH of the wastewater and reaction time all affected the H_2_ production and pollutant removal levels. At the optimum conditions found, approximately 228 μmol of H_2_ were produced with COD, BOD and oil and grease removal levels of 13.2%, 89.6% and 67.7%, respectively, and with a light conversion efficiency of 6.78%. The pH and BOD levels were reduced to within the acceptable Thai standard, but the levels of COD, oil and grease, TDS and TSS were still greater than the acceptable values.

## Figures and Tables

**Table 1:** Properties of the fresh, acid-pretreated and UV-TiO_2_-treated biodiesel wastewater. COD, chemical oxygen demand; BOD, biological oxygen demand.

Property	Thai Standard	Fresh Wastewater	Pretreated Wastewater ^a^	Treated Wastewater ^b^
pH	5.5–9.0	4.07–4.12	1.12–2.22	8.75–8.78
Soap (wt %)	-	50.68–51.75	31.05–33.33	0.09–1.07
FFA (wt %)	-	1.09–1.23	7.63–7.82	0.02–0.04
Glycerol (wt %)	-	0.85–0.86	0.98–1.11	N/D
COD (mg/L)	≤400	118,220–146,878	60,815–96,600	24,738–24,911
BOD (mg/L)	≤60	620–1193	210–460	9.0–13.6
Oil and grease (mg/L)	≤15	660–1885	336–1338	205–243
TDS (mg/L)	≤3000	7392–13,568	11,496–12,584	7710–9100
TSS	≤150	528–628	128–312	140–190

^a^ Pretreated by H_2_SO_4_ addition to a pH of around 2, as reported [14]. ^b^ Treated by photocatalytic oxidation with 3.3-fold dilution, initial pH of 6.0, TiO_2_ dosage of 4.0 g/L, UV light intensity of 4.79 mW/cm^2^ and irradiation time of 4 h.
